# Discovery of a new candidate drug to overcome cabazitaxel-resistant gene signature in castration-resistant prostate cancer by in silico screening

**DOI:** 10.1038/s41391-021-00426-0

**Published:** 2021-09-30

**Authors:** Hiroshi Hongo, Takeo Kosaka, Yoko Suzuki, Mototsugu Oya

**Affiliations:** Department of Urology, Keio University School of Medicine, 35 Shinanomachi, Shinjuku-ku, Tokyo, 160-8582 Japan

**Keywords:** Cancer therapy, Prostate cancer

## Abstract

**Background:**

The taxane cabazitaxel (CBZ) is a promising treatment for docetaxel-resistant castration-resistant prostate cancer (CRPC). However, the survival benefit with CBZ for patients with CRPC is limited. This study used screening tests for candidate drugs targeting CBZ-resistant-related gene expression and identified pimozide as a potential candidate for overcoming CBZ resistance in CRPC.

**Methods:**

We established CBZ-resistant cell lines, DU145CR and PC3CR by incubating DU145 cells and PC3 cells with gradually increasing concentrations of CBZ. We performed in silico drug screening for candidate drugs that could reprogram the gene expression signature of a CBZ-resistant prostate cancer cells using a Connectivity Map. The in vivo effect of the drug combination was tested in xenograft mice models.

**Results:**

We identified pimozide as a promising candidate drug for CBZ-resistant CRPC. Pimozide had a significant antitumor effect on DU145CR cells. Moreover, combination treatment with pimozide and CBZ had a synergic effect for DU145CR cells in vitro and in vivo. Microarray analysis identified *AURKB* and *KIF20A* as potential targets of pimozide in CBZ-resistant CRPC. DU145CR had significantly higher *AURKB* and *KIF20A* expression compared with a non-CBZ-resistant cell line. Inhibition of *AURKB* and *KIF20A* had an antitumor effect in DU145CR xenograft tumors. Higher expression of *AURKB* and *KIF20A* was a poor prognostic factor of TGCA prostate cancer cohort. CBZ-resistant prostate cancer tissues in our institution had higher *AURKB* and *KIF20A* expression.

**Conclusions:**

Pimozide appears to be a promising drug to overcome CBZ resistance in CRPC by targeting *AURKB* and *KIF20A*.

## Introduction

Prostate cancer is the most prevalent cancer among American men and the second leading cause of cancer death [1, 2]. Although metastatic prostate cancer initially responds well to androgen deprivation, most patients acquire resistance to this therapy, developing castration-resistant prostate cancer (CRPC). Cabazitaxel (CBZ) is a second-generation taxane indicated for the treatment of metastatic CRPC previously treated with a docetaxel-containing regimen [3]. However, the survival benefit of CBZ for patients with CRPC is limited to only a few months [4]. Because CBZ-resistant CRPC has such a poor prognosis, establishing novel treatments for CBZ-resistant CRPC is an as yet unmet medical need.

We previously reported the establishment of CBZ-resistant CRPC cell lines [5]. The CBZ resistance mechanism reflects various biological processes associated with complex gene expression networks. Many current computational strategies take advantage of shared similarities among drugs, including their molecular activity, drug-induced side effects, or chemical structures. Instead of searching for signaling pathways or gene products with potential as therapeutic targets from the lists of CBZ resistance-associated genes, we directly submitted the gene lists to the Connectivity Map data base [6]. This public resource contains gene signatures obtained after treating cells with various chemical compounds. It has pattern-matching tools to investigate similarities between these reported signatures and gene lists uploaded by the user. In this study, we performed this type of in silico screening test for candidate drugs targeting CBZ resistance and identified pimozide (PZD) as a potential candidate to overcome CBZ resistance in CRPC. We further investigated its possible mechanism of action to clarify which gene products are the targets of this drug.

## Materials and methods

### Reagents

We used anti-AURKB rabbit monoclonal antibody (Abcam, Cambridge, UK), anti-KIF20A rabbit polyclonal antibody (Bethyl Laboratories, Montgomery, TX, USA), and anti-KIF20A (Santa Cruz Biotechnology, Santa Cruz, CA, USA) and β-actin (Sigma-Aldrich, St. Louis, MO, USA) mouse monoclonal antibody. WST-1 reagents (Takara Bio, Kyoto, Japan) were used for cell viability assays.

### Cell lines and culture

Prostate cancer cell lines LNCaP, DU145, and PC3 were obtained from the American Type Culture Collection. DU145CR was established by incubating DU145 cells with gradually increasing concentrations of CBZ [5]. DU145 and DU145CR were routinely maintained in RPMI 1640 (Invitrogen, Carlsbad, CA, USA) supplemented with 10% fetal bovine serum (Dainippon Pharmaceutical, Tokyo, Japan) at 37 °C in a humidified 5% CO_2_ atmosphere.

### WST cell viability assay

DU145 and DU145CR cells were seeded on 96-well plates, allowed to attach for 24 h, and then treated with varying concentrations of CBZ, PZD, protriptyline, pyrvinium (Sigma-Aldrich), and syrosingopine (WAKO, Tokyo, Japan) for 48 h. At the end of the incubation period, WST reagents were added to each well and the cells were incubated for 1 h. Cell viability was estimated by colorimetry by reading the color intensity in a plate reader at 570 nm.

### Murine Xenograft prostate cancer model

Five- to seven-week-old male athymic nude BALB-C mice were castrated by scrotal incision under anesthesia to create a xenograft model. DU145 and DU145CR cells (2 × 10^6^ cells) suspended in 100 μL Matrigel (Becton Dickinson Labware, Lincoln Park, NJ, USA) were subcutaneously inoculated into the mice. The tumors were measured every 4 days. When the mean tumor volume reached ~100 mm^3^, the mice were randomly assigned to one of four groups with eight mice per group: an untreated control group or three treatment groups treated with intraperitoneal CBZ only (10 mg/kg), peroral PZD only (7.5 mg/kg/day), or intraperitoneal CBZ (10 mg/kg) combined with peroral PZD (7.5 mg/kg/day). On day 13, the mice were anaesthetized with sevoflurane (WAKO, Tokyo, Japan) and killed by cervical dislocation. The subcutaneous tumors were harvested. Animal care was performed in accordance with the Keio University guidelines for animal experiments. The study was conducted according to the Animal Research Reporting In Vivo Experiments requirements (Supplementary Methods).

### Immunohistochemistry

We immunostained 4-µm sections of formalin-fixed, paraffin-embedded xenograft tumors. After antigen retrieval with citric acid (pH 6.0), endogenous peroxidase activity was blocked with 1% hydrogen peroxide. Primary antibodies (monoclonal anti-Ki67 antibody, dilution 1:200, monoclonal anti-AURKB antibody, dilution 1:200, and polyclonal anti-KIF20A antibody, dilution 1:200) were applied and incubated with secondary antibodies conjugated to peroxidase-labeled dextran polymer. The immunoreaction was visualized using diaminobenzidine and counterstaining with 10% hematoxylin. The percent of cancer cells with nuclei stained for Ki67 (the Ki67 index) was calculated for each section based on more than 1000 cell nuclei. The histoscores for AURKB and KIF20A were calculated on the basis of the mean percentage intensity (range 0–300). Immunohistochemistry was performed according to previous reports [7–10].

Apoptosis was measured by a TUNEL assay using an in situ apoptosis detection kit (Takara Bio, Kyoto, Japan). Control slides from the apoptosis detection kit were used as the positive control, and slides without terminal deoxynucleotidyl transferase enzyme were used as the negative control. The average number of stained cells was counted and the apoptosis index calculated as the average number in five areas of a 400× field.

### Microarray gene expression analysis

Comprehensive analysis of gene expression in DU145 and DU145CR was performed using microarray. Total RNA was isolated from the cell lines using an RNeasy Mini kit (Qiagen, Valencia, CA, USA). Gene expression profiles were determined using the Affymetrix GeneChip Human Gene 1.0 ST array according to the manufacturer’s instructions. After generating single-stranded cDNA, fragmentation and sense-strand cDNA labeling was performed with an Affymetrix GeneChip WT Terminal Labeling Kit (Affymetrix, Santa Clara, CA, USA) according to the manufacturer’s protocol. After hybridization, a GeneChip Fluidics Station 450 (Affymetrix) was used to wash the arrays. Scanning was performed with a GeneChip Scanner 3000 7G (Affymetrix). The raw intensity data from scanned images of the microarrays were preprocessed using Affymetrix Expression Console software. Expression intensities were stored as cell intensity (CEL) files, and the CEL files were normalized with the robust multichip average method. To identify compounds that could reprogram the CBZ resistance-related genetic network, the CBZ resistance signature was estimated by calculating the twofold gene changes from DU145 to DU145CR, after which the probe list of the CBZ resistance signature was entered into the Connectivity Map (http://www.broadinstitute.org/cmap/). According to the Connectivity Map system [6, 11–20], the top 500 upregulated and downregulated probes compatible with the HG-U133A platform were used. The threshold of significance for the candidate compounds was set at *p* < 0.05. This microarray dataset has been approved by the Gene Expression Omnibus (http://www.ncbi.nlm.gov/geo/); its accession number is GSE110107.

### Cell extracts and western blots

Whole cell extracts were obtained using RIPA buffer composed of 50 mM tris-HCl (pH 7.5), 150 mM NaCl, 1% NP-40, 0.5% deoxycholate, 0.1% sodium dodecyl sulfate, and protease inhibitors. For western blots, 50 mg of total protein was separated by sodium dodecyl sulfate-polyacrylamide gel electrophoresis on 12.5% acrylamide gel and transferred to a nitrocellulose membrane. Blots were incubated with peroxidase-labeled secondary antibody (Dako). Signals were detected using enhanced chemiluminescence reagents with a detection system (Pierce ECL Plus Western Blotting Detection System, Thermo Scientific, MA, USA), and signal intensity was quantified using a LAS 3000 system (Fujifilm, Tokyo, Japan). Human overexpression lysates of AURKB and KIF20A were obtained from OriGene Technologies, Rockville, MD, USA.

### Real-time quantitative PCR

Total RNA was isolated using an RNeasy Mini kit (Qiagen, Hilden, Germany), and the quantity and quality were evaluated by spectrophotometry. Reverse transcription of RNA to cDNA was conducted using a PrimeScript RT reagent kit with gDNA Eraser (Takara Bio, Kyoto, Japan). The reaction mixture (1 μL) was then used as a template in a TaqMan Fast real-time quantitative PCR assay using TaqMan Universal PCR Master Mix and the CFX96 Touch Real-Time PCR Detection System (Bio-Rad Laboratories, Hercules, CA, USA). The primers and TaqMan probe sets (TaqMan Gene Expression Assays) for *AURKB* (Hs00945858_g1), *KIF20A* (Hs00993573_m1), and human GAPDH endogenous control (Hs99999903_m1) were purchased from Applied Biosystems (sequences not disclosed). The cycling conditions were 50 °C for 10 min and 95 °C for 10 min followed by 40 cycles at 95 °C for 15 s and at 60 °C for 1 min.

### Flow cytometry

DU145 and DU145CR cells were incubated for 24 h with or without 10 μM PZD. They were harvested by exposing them to 0.25% trypsin-EDTA solution for 5 min, centrifuging and washing in phosphate-buffered saline (PBS), fixing in formaldehyde for 15 min, and then incubating in 100% methanol overnight at −20 °C. After overnight incubation, the cells were centrifuged, washed once again in PBS, and incubated in monoclonal anti-AURKB antibody, dilution 1:200, and monoclonal anti-KIF20A antibody, dilution 1:100. The cells were then incubated in anti-rabbit Alexa 555 antibody (dilution 1:200) and anti-mouse Alexa 488 (dilution 1:200). After resuspending the cells in Propidium Iodide/RNase Staining Solution (Cell Signaling Technology, Danvers, MA, USA), cell distribution was analyzed using a FlowSight Imaging Flow Cytometer (Merck Millipore, Billerica, MA, USA).

### Small interfering RNA

The following predesigned duplex siRNA (Applied Biosystems) was used to knock down the *AURKB* or *KIF20A* expression in DU145 or DU145CR. The sense sequences of the siRNAs were as follows: si-AURKB, 5ʹ-UCGUCAAGGUGGACCUAAA-3ʹ and si-KIF20A, 5′-GGAACAUAGUCUUCAGGUA-3′. Cells were transiently transfected with 20 nmol of the respective siRNAs using Lipofectamine RNAiMAX (Life Technologies, Carlsbad, CA). After 12 h, siRNA was removed by replacing the culture medium with fresh RPMI 1640 containing 1% FBS, and the cells were further incubated for 36 h. A mock-transfected control was prepared using the transfection reagent only.

### Overexpression of AURKB and KIF20A

*AURKB* or *KIF20A* upregulation in DU145 or DU145CR was performed using the *AURKB* and *KIF20A* plasmids. These plasmids were stably transfected into DU145 cells using the Xtreme GENE HP DNA transfection reagent (Roche, San Francisco, CA, USA) according to the manufacturer’s instructions. The empty pBApo-CMV Neo vector (Takara Bio Inc, Shiga, Japan) was also transfected into DU145 as control vector.

### Data analysis of prostate cancer cohorts

Recurrence-free survival and mRNA expression of AURKB and KIF20A in the cancer genome atlas (TCGA) prostate cancer dataset [21], which included primary prostate cancer cases, were extracted from cBioPortal (http://www.cbioportal.org/). The prognostic significance of *AURKB* and *KIF20A* expression was examined using Kaplan–Meier survival analysis, and recurrence-free survival outcome was compared by log-rank tests. High expression of *AURKB* or *KIF20A* was defined as an expression >1 standard deviation from the mean. Information on chemotherapy and mRNA expression of *AURKB* and *KIF20A* in the Fred Hutchinson (FH) Cancer Research Center prostate cancer dataset, [22] which included metastatic prostate cancer cases, was also extracted from cBioPortal (http://www.cbioportal.org/). Differences in *AURKB* and *KIF20A* expression between prechemotherapy and postchemotherapy data were analyzed using a *t*-test.

Biopsy specimens from three CBZ-resistant CRPC patients at Keio University Hospital were used for this study. The characteristics of the patients are presented in Supplementary Table 1. Immunohistochemical staining of *AURKB* and *KIF20A* on formalin-fixed paraffin-embedded sections was performed as described above.

### Statistical analysis

Experiments were replicated three or more times. Statistical analysis was performed using the t-test and Tukey–Kramer method for multiple comparison test, with *p* < 0.05 considered significant.

## Results

### Effect of PZD on cabazitaxel-resistant prostate cancer

**Fig. 1:**
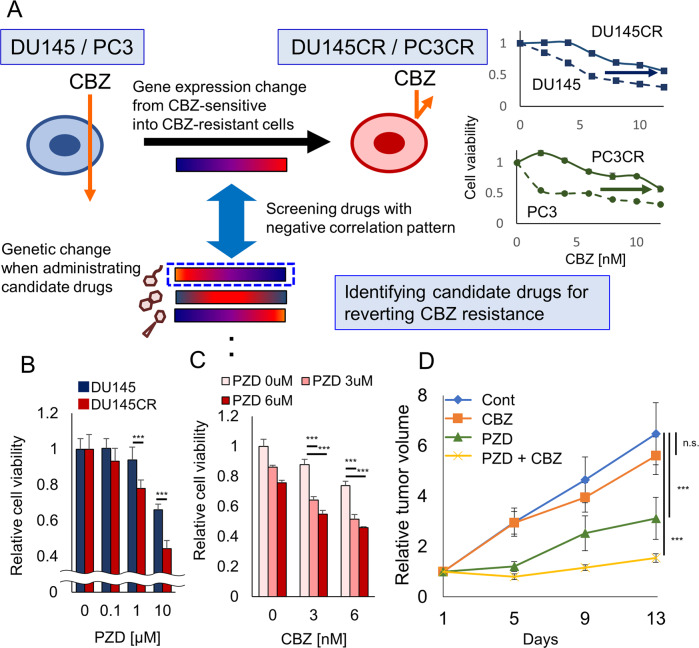
Drug screening for overcoming cabazitaxel-resistant prostate cancer. **A** Schema of drug screening for overcoming cabazitaxel resistance in prostate cancer using a Connectivity Map. **B** In the WST assay, the relative viability of CBZ-resistant DU145CR cells treated with various doses of PZD was significantly lesser than that of CBZ-sensitive DU145 cells. **C** Cell viability of DU145CR with various doses of PZD and CBZ. **D** Tumor growth over time of DU145CR xenograft tumors in castrated male nude mice during treatment with 10 mg/kg of CBZ, 7.5 mg/kg/day PZD, CBD + PZE, or no treatment (Cont).

Using our previously established CBZ-resistant cell line, DU145CR [5], we analyzed the gene expression changes from DU145 to DU145CR cells using microarray analysis in order to perform in silico drug screening using the Connectivity Map [6] for candidate drugs reprogramming the gene expression signature in DU145CR (Fig. 1A). We tested the antitumor effect of the listed candidate drugs (Supplementary Table 2) for DU145 and DU145CR in vitro. Among these, protriptyline, pyrvinium, or syrosingopine did not significantly inhibit DU145CR proliferation (Supplementary Fig. 1A–C), while PZD did (Fig. 1B). Next, we tested the efficacy of treating DU145CR cells with CBZ combined with PZD (Fig. 1C). The combination index for PZD co-administration was 0.63, suggesting that this combination has a synergistic effect. PZD and CBZ did not have a synergic effect on DU145 (Connectivity index 0.93) (Supplementary Fig. 2). PZD also had an antitumor effect on PC3CR in vitro (Supplementary Fig. 3A, B). Protriptyline, pyrvinium, and syrosingopine had no synergic effect with CBZ (Supplementary Fig. 1D–F). There are some limitations to the CBZ-resistant models used in our study because DU145CR and PC3CR cells lack androgen receptors (ARs). Most of the patients with CRPC still express AR. We established a CBZ-resistant AR-positive prostate cancer model, LNKO6CR, which was derived by incubating LNCaP under androgen ablation conditions for 6 months [23] and subsequently incubating with gradually increasing CBZ concentrations for 12 months (Supplementary Fig. 4A). PZD was also effective on LNKO6CR (Supplementary Fig. 4B). To test the efficacy of PZD in vivo, we administered CBZ and PZD to mice with DU145CR xenografts. Although intraperitoneal treatment of 10 mg/kg of CBZ did not suppress tumor growth, daily oral treatment with 7.5 mg/kg of PZD had a significant antitumor effect on DU145CR tumors (Fig. 1D). The combination of PZD and CBZ had a synergistic effect (Fig. 1D). Similar results were obtained in PC3CR xenograft tumors (Supplementary Fig. 2C). The Ki67 index in the PZD monotherapy and combined treatment groups were significantly lower than that in the control and CBZ monotherapy groups (Supplementary Fig. 5A, B). Similarly, PZD monotherapy and combination treatment had a significantly higher apoptosis index than the control and CBZ monotherapy groups (Supplementary Fig. 5C, D).

### Molecular mechanism of action of PZD

**Fig. 2:**
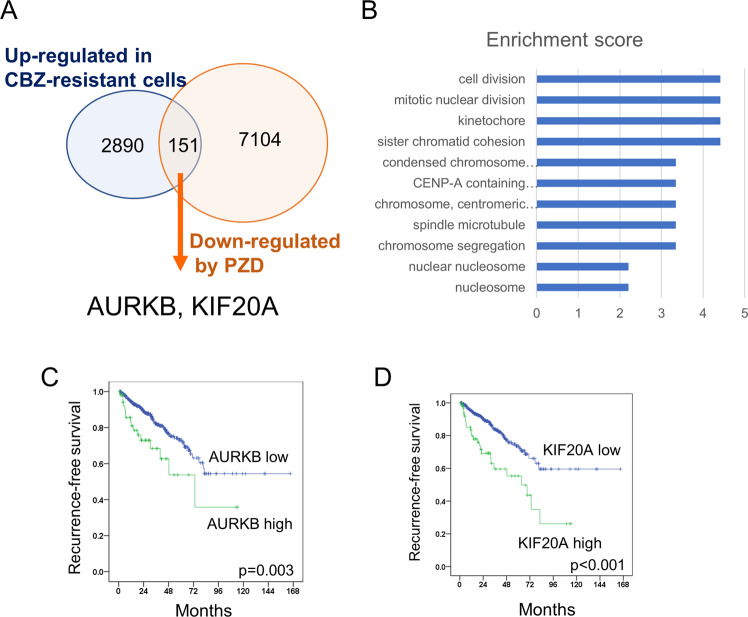
AURKB and KIF20A as therapeutic targets of pimozide (PZD). **A** Venn diagram of genes upregulated in CBZ-resistant DU145CR and downregulated by PZD. **B** DAVID functional annotation clustering revealed genes related to cell division, chromatin cohesion, and kinetochore were downregulated by PZD. **C** Kaplan–Meier curves of recurrence-free survival in The Cancer Genome Atlas prostate cancer cohort by high or low *AURKB* expression. **D** Kaplan–Meier curves of recurrence-free survival according to *KIF20A* expression in The Cancer Genome Atlas prostate cancer cohort.

**Fig. 3:**
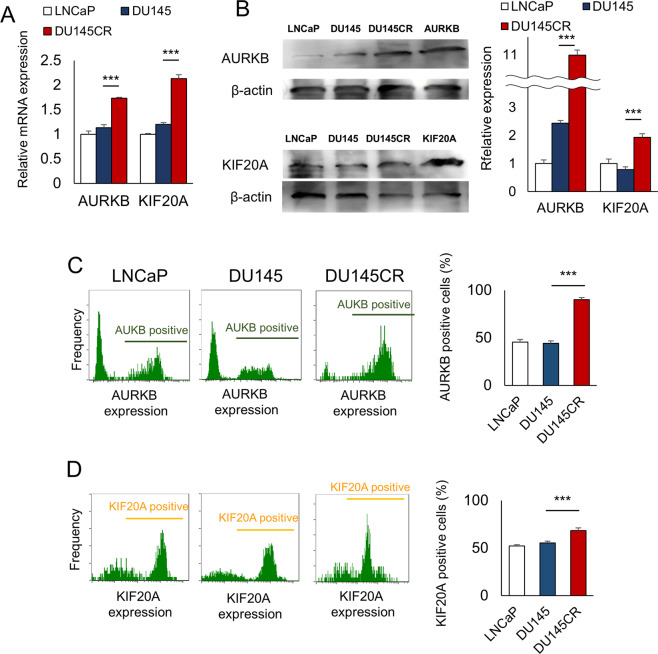
AURKB / KIF20A expression in cabazitaxel-resistant prostate cancer cells. **A** AURKB and KIF20A mRNA expression in CBZ-sensitive DU145 and in DU145CR cells. **B** AURKB or KIF20A protein expression in LNCaP, DU145, DU145CR cells and overexpression lysates of AURKB or KIF20A. **C** Flow cytometry indicating the percentage of AURKB-positive cells is increased in DU145CR vs. DU145 cells. **D** KIF20A-positive cells are also increased in DU145CR cells.

To investigate the potential molecular targets of PZD, we analyzed the changes in the gene expression induced by PZD using microarrays. A total of 7104 genes in DU145CR cells were downregulated by PZD, whereas 2890 genes were upregulated in DU145CR compared with DU145. The Venn diagram in Fig. 2A shows the genes in DU145CR upregulated and downregulated by PZD, which could be potential targets of PZD in CBZ-resistant CRPC. We investigated the function of the 151 genes downregulated by PZD. DAVID functional annotation clustering revealed genes related to cell division, chromatin cohesion, and kinetochore were downregulated by PZD (Fig. 2B). Next, we investigated the prognostic significance of the expression of these 151 genes in prostate cancer using the TCGA cohort data. Among these genes, expressions of *AURKB* and *KIF20A* was a significantly poor prognostic factors for prostate cancer recurrence, and we focused on these two genes (Fig. 2C, D). In quantitative PCR (Fig. 3A) and western blotting (Fig. 3B), DU145CR cells demonstrated significantly higher AURKB and KIF20A expression at the protein and mRNA expression levels compared with DU145 cells. PC3CR also had higher *AURKB* and *KIF20A* expression than PC3 (Supplementary Fig. 6). Flow cytometry revealed that the numbers of AURKB-positive cells and KIF20A-positive cells were higher in DU145CR than those in DU145 cells (Fig. 3C, D). Immunohistochemistry yielded similar findings in sections of the DU145 and DU145CR xenograft tumors (Supplementary Fig. 7A, B).

### AURKB as a therapeutic target in cabazitaxel-resistant prostate cancer

**Fig. 4:**
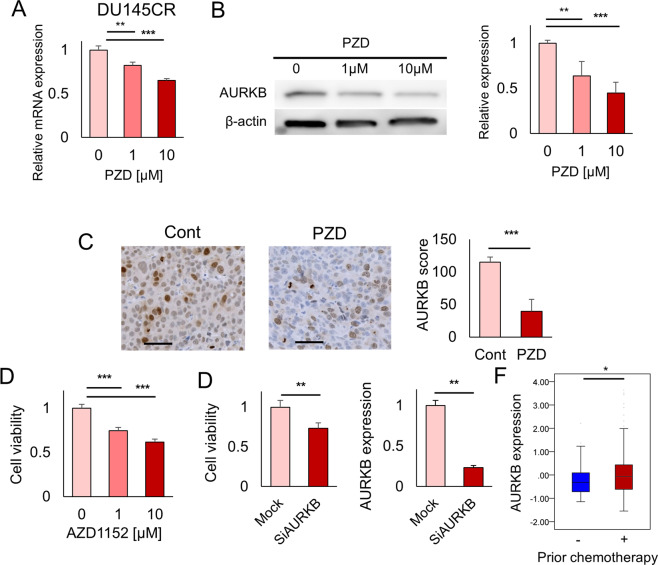
AURKB as a therapeutic target in cabazitaxel (CBZ)-resistant prostate cancer. **A** Pimozide (PZD) suppressed *AURKB* mRNA expression in CBZ-resistant DU145CR cells. **B** PZD suppressed AURKB protein expression in DU145CR cells. **C** AURKB expression in DU145CR xenograft tumors was suppressed by PZD administration. **D** In the cell viability assay, the AURKB inhibitor AZD1152 had an antitumor effect in DU145CR cells. **E** AURKB knockdown by siRNA inhibited the proliferation of DU145CR cells. **F** In the Fred Hutchinson Cancer Research Center prostate cancer dataset cohort, *AURKB* expression was significantly upregulated in postchemotherapy prostate cancer tissues.

We investigated whether AURKB was a potential target for the treatment of CBZ-resistant prostate cancer. In both quantitative PCR (Fig. 4A) and western blotting analysis (Fig. 4B), PZD inhibited AURKB expression. Protriptyline, pyrvinium, and syrosingopine did not suppress AURKB expression. (Supplementary Fig. 1G). Flow cytometry revealed that the numbers of AURKB-positive cells were decreased after PZD administration in DU145CR cells (Supplementary Fig. 8A). AURKB expression in DU145CR xenograft tumors was also inhibited by PZD administration (Fig. 4C). In the cell viability assay, the AURKB inhibitor AZD1152 or AURKB knockdown had an antitumor effect (Fig. 4D, E). AURKB inhibition also had an antitumor effect in PC3CR (Supplementary Fig. 9A). We tested the contribution of AURKB overexpression to CBZ resistance. AURKB overexpression decreased the CBZ sensitivity of DU145 cells (Supplementary Fig. 10A). These results suggested that the *AURKB* gene contributed to CBZ resistance. Prognosis analysis using the TCGA prostate cancer dataset revealed that a higher *AURKB* expression was a poor prognostic factor for recurrence-free survival (Fig. 2C). In the FH cohort, which included metastatic prostate cancer cases, *AURKB* expression was significantly upregulated in postchemotherapy prostate cancer tissues (Fig. 4F). In addition, we analyzed AURKB expression in CBZ-resistant CRPC tissues from patients in our institution by immunohistochemistry, which revealed high AURKB expression (Supplementary Fig. 11).

### KIF20A as a therapeutic target in cabazitaxel-resistant prostate cancer

**Fig. 5:**
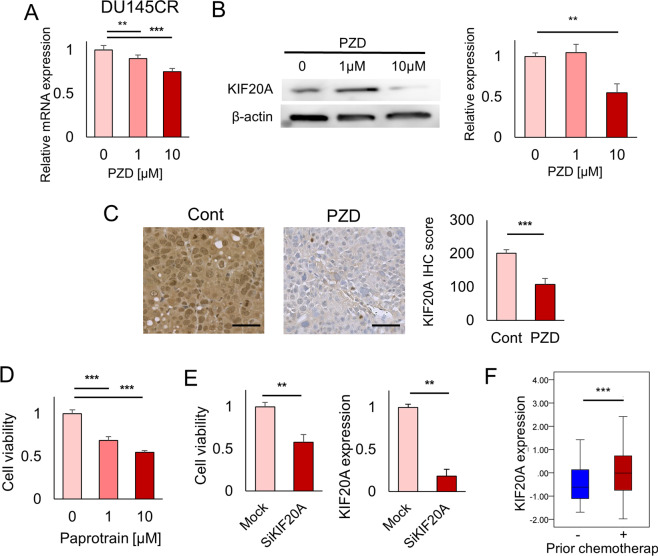
KIF20A as a therapeutic target in cabazitaxel-resistant prostate cancer. **A** Pimozide (PZD) suppressed KIF20A mRNA expression in cabazitaxel-resistant DU145CR cells. **B** PZD suppressed KIF20A protein expression in DU145CR cells. **C** KIF20A expression in DU145CR xenograft tumors was suppressed by PZD administration. **D** The KIF20A inhibitor paprotrain had an antitumor effect on DU145CR cells in a cell viability assay. **E** KIF20A knockdown by siRNA also inhibited DU145CR cell proliferation. **F** *KIF20A* expression was significantly upregulated in postchemotherapy prostate cancer tissues in the Fred Hutchinson Cancer Research Center prostate cancer cohort.

We also investigated the role of KIF20A in CBZ-resistant prostate cancer. In quantitative PCR, western blotting, and flow cytometry, PZD suppressed KIF20A in DU145CR cells (Fig. 5A, C and Supplementary Fig. 8B). Protriptyline, pyrvinium, and syrosingopine did not suppress KIF20A expression in DU145CR (Supplementary Fig. 1G). KIF20A expression in DU145CR xenograft tumors was also inhibited by PZD administration (Fig. 5D). The KIF20A inhibitor paprotrain or KIF20A knockdown had an antitumor effect in DU145CR cells (Fig. 5E, F). KIF20A inhibition also had an antitumor effect in PC3CR (Supplementary Fig. 9B). We tested the contribution of KIF20A overexpression in CBZ resistance. KIF20A overexpression decreased the CBZ sensitivity of DU145 cells (Supplementary Fig. 10B). These results suggested that the *KIF20A* gene contributed to CBZ resistance. Prognosis analysis revealed that higher *KIF20A* expression was a poor prognostic factor for recurrence-free survival (Fig. 2D). In the FH cohort, *KIF20A* expression was significantly upregulated in postchemotherapy prostate cancer tissues (Fig. 5G). Immunohistochemistry of CBZ-resistant CRPC tissues in our institution revealed high expression of KIF20A (Supplementary Fig. 11).

## Discussion

Drug resistance, including docetaxel, is often caused by upregulated P-glycoprotein (P-gp) levels. However, the mechanism of CBZ resistance is still unclear. We have previously reported in Cancer Science 2018 that our CBZ-resistant cell lines did not have upregulated P-gp levels as compared with the parental CBZ-sensitive cell lines [5]. In this study, we used bioinfomatics analysis to identify a possible new indication for PZD, namely, to overcome CBZ resistance in CRPC. PZD is an orally active antipsychotic drug of the diphenylbutylpiperidine class that inhibits dopaminergic receptors. Although PZD has been reported to have antitumor efficacy in pancreatic cancer [24], there are no previous reports indicating AURKB or KIF20A inhibition as the possible mechanism for its effects. PZD, approved for human use as an orally active antipsychotic agent in 1980s, is thought to be a promising novel drug for CBZ-resistant CRPC.

Currently, substantial resources are needed for drug discovery and development with, for example, high-throughput screening. In silico drug screening of drug databases enables less costly drug discovery. Because candidate drugs in the databases are already approved for human use, they will likely have fewer side effects than the newly developed agents. The Connectivity Map launched by Justin Lamb et al. [6] is a database of gene expression changes caused by chemical compounds or genetic manipulation. Previous studies have suggested that this tool can be applied for screening drugs that reverse the genetic changes induced by various diseases, including refractory cancer [12–16]. We also identified ribavirin as a drug capable of reprogramming docetaxel resistance in CRPC by in silico screening using the Connectivity Map [20]. On the basis of the same concept that targets gene expression profiles, we performed in silico screening tests in this study to find candidate drugs and their possible targets in CBZ resistance-related gene networks　(Supplementary Fig. 10).

The aurora kinase family plays an important role in several aspects of cell proliferation in human cells. Aurora kinase A (AURKA) is one of the key molecules involved in spindle formation. It recruits γ-tubulin and TACC/MAP215, which stabilize the mitotic spindle. In prostate cancer, AURKA is reported to contribute to the epithelial–mesenchymal transition and neuroendocrine differentiation [25]. Although AURKB had been thought to be a tumor suppressor gene, some studies have found that the upregulation of AURKB is a risk factor for cancer development [26, 27]. Higher AURKB expression was reported in prostate cancers with a high Gleason grade [28]. AURKB has been suggested to relate to androgen signaling in transgenic adenocarcinoma of the mouse prostate [29]. AURKB has also been reported as a factor related to chemotherapy resistance in other cancers [30, 31]. *AURKB* mRNA expression is thought to modulate paclitaxel resistance in lung cancer [32]. However, there are no reports that describe how AURKB contributes to CBZ resistance in prostate cancer. In this study, a CBZ-resistant CRPC cell line had higher AURKB expression, and PZD inhibited that expression. These results suggest the AURKB is a potential therapeutic target in CBZ-resistant CRPC.

We obtained similar experimental findings for KIF20A, a member of the kinesin superfamily of proteins. In cell cycle regulation, they are known to mediate spindle formation and cytokinesis [33]. KIF20A also contributes to cytokinesis in collaboration with AURKB activity [34]. KIF20A is reportedly associated with paclitaxel resistance in breast cancer and could therefore be a therapeutic target for refractory breast cancer [35]. Other kinesin superfamily proteins, such as KIF2C [36] or KIF3A [37] are associated with prostate tumor progression. However, the significance of KIF20A expression in tumor progression or chemoresistance in prostate cancer has been unclear. In our study, as with AURKB, KIF20A expression was high in CBZ-resistant CRPC cell lines, xenografts, and human tissues. The expression level was downregulated in each case by treatment with PZD. Thus, KIF20A may also be a worthy target in CBZ-resistant CRPC. Certainly, prospective cohort studies are needed to validate the significance of the AURKB/KIF20A expression for CBZ resistance. However, our results suggested that AURKB and KIF20A were associated with the acquired CBZ resistance.

In conclusion, PZD appears to be a promising drug for overcoming CBZ resistance in CRPC by targeting AURKB and KIF20A.

## Supplementary information

Supplementary Legends

Supplementary Figures

Supplementary Table 1

Supplementary Table 2

Supplementary Methods

## Data Availability

The datasets used and/or analyzed during the current study are available from the corresponding authors on reasonable request.
