# Supplementary material for: Discovery of a new candidate drug to overcome cabazitaxel-resistant gene signature in castration-resistant prostate cancer by in silico screening
Source: Prostate Cancer Prostatic Dis. 2021 Sep 30;26(1):59–66. doi: 10.1038/s41391-021-00426-0 (PMC10023558; doi:10.1038/s41391-021-00426-0)
Supplement: Supplementary file 1 — Supplementary Legends [file 41391_2021_426_MOESM1_ESM.docx]

**Supplementary Legends**

Supplementary Figure 1. *In vitro* screening of candidate drugs to overcome CBZ resistance in castration-resistant prostate cancer.

A-C, Protriptyline (A), pyrvinium (B), nor syrosingopine (C) did not inhibit the proliferation of cabazitaxel-sensitive DU145 or cabazitaxel-resistant DU145CR cells in cell viability assays. D-E, Protriptyline (D), pyrvinium (E), nor syrosingopine (F) did not have synergic effect on DU145CR. G, Protriptyline, pyrvinium, nor syrosingopine did not suppress AURKB nor KIF20A expression in DU145CR.

Supplementary Figure 2. Combined administration of PZD and CBZ for DU145. PZD and CBZ did not have synergic effect on DU145.

Supplementary Figure 3. Antitumor effect of PZD in PC3CR. A, In the WST assay, the relative viability of CBZ-resistant PC3CR cells treated with various doses of PZD was significantly lesser than that of CBZ-sensitive PC3 cells. B, Cell viability of PC3CR with various doses of PZD + CBZ. C, Tumor growth over time of PC3CR xenograft tumors in castrated male nude mice during treatment with 10 mg/kg of CBZ, 7.5 mg/kg/day PZD, CBD + PZE, or no treatment (Cont).

Supplementary Figure 4. Antitumor effect of PZD for AR-positive CBZ-resistant prostate cancer cells. A, LNKO6CR had significantly lower sensitivity to CBZ than LNKO6. B, In the WST assay, the relative viability of CBZ-resistant LNKO6CR cells treated with various doses of PZD was significantly lesser than that of CBZ-sensitive LNKO6 cells.

Supplementary Figure 5. Ki67 and TUNNEL staining in DU145CR xenograft tumors. A, Representative immunohistochemical staining for Ki67 in DU145CR xenograft tumors. B, Significant decrease in the Ki67 index is shown for CBZ + PZD compared with other groups. C, Representative TUNEL staining in DU145CR xenograft tumors. D, A significant increase in the apoptosis index was noted for PZD + CBZ compared with other treatments.

Supplementary Figure 6. Upregulation of AURKB and KIF20A in PC3CR. A, AURKB and KIF20A mRNA expression in CBZ-sensitive PC3 and in PC3CR cells. B, AURKB and KIF20A protein expression in PC3 and PC3CR cells.

Supplementary Figure 7. AURKB and KIF20A expression in a murine DU145 or DU145CR xenograft model. A, AURKB expression is upregulated in cabazitaxel (CBZ)-resistant DU145CR tumors compared with CBZ-sensitive DU145 tumors. B, KIF20A expression is upregulated in DU145CR tumors compared with DU145 tumors.

Supplementary Figure 8. Flow-cytometry of DU145CR cells. Flow cytometry demonstrated a decreased percentage of ARUKB (A) or KIF20A (B)-positive DU145CR cells after PZD administration compared with control (Cont) cells.

Supplementary Figure 9. Antitumor effect of AURKB or KIF20A inhibition for PC3CR. A, In the cell viability assay, the AURKB inhibitor AZD1152 had an antitumor effect in PC3CR cells. B, The KIF20A inhibitor paprotrain had an antitumor effect on PC3CR cells in a cell viability assay.

Supplementary Figure 10. Contribution of AURKB/KIF20A overexpression to CBZ resistance in prostate cancer cells. In the WST assay, *AURKB* (A, B) or *KIF20A* (C, D) overexpression decreased the CBZ sensitivity of the DU145 cells

Supplementary Figure 11. *AURKB* and *KIF20A* expression in cabazitaxel (CBZ)-resistant castration-resistant prostate cancer (CRPC) tissues from human tumors. A, Representative immunohistochemical staining for AURKB in clinical CRPC tissues. B, The AURKB IHC score in clinical CRPC tissue. C, Representative immunohistochemical staining for KIF20A in clinical CRPC tissues. D, KIF20A IHC score in clinical CRPC tissues.

Supplementary Figure 12. Schematic representation of the mechanism of antitumor action of PZD on cabazitaxel-resistant prostate cancer.

Supplementary Table 1. Patients’ characteristics

Supplementary Table 2. Candidate compounds to overcome cabazitaxel resistance in castration-resistant prostate cancer.

Supplementary Methods. Animal studies in compliance with the ARRIVE guidelines.
