# Supplementary material for: Discovery of a new candidate drug to overcome cabazitaxel-resistant gene signature in castration-resistant prostate cancer by in silico screening
Source: Prostate Cancer Prostatic Dis. 2021 Sep 30;26(1):59–66. doi: 10.1038/s41391-021-00426-0 (PMC10023558; doi:10.1038/s41391-021-00426-0)

Supplementary Figure 1. *In vitro* screening of candidate drugs overcoming CBZ-resistant CRPC

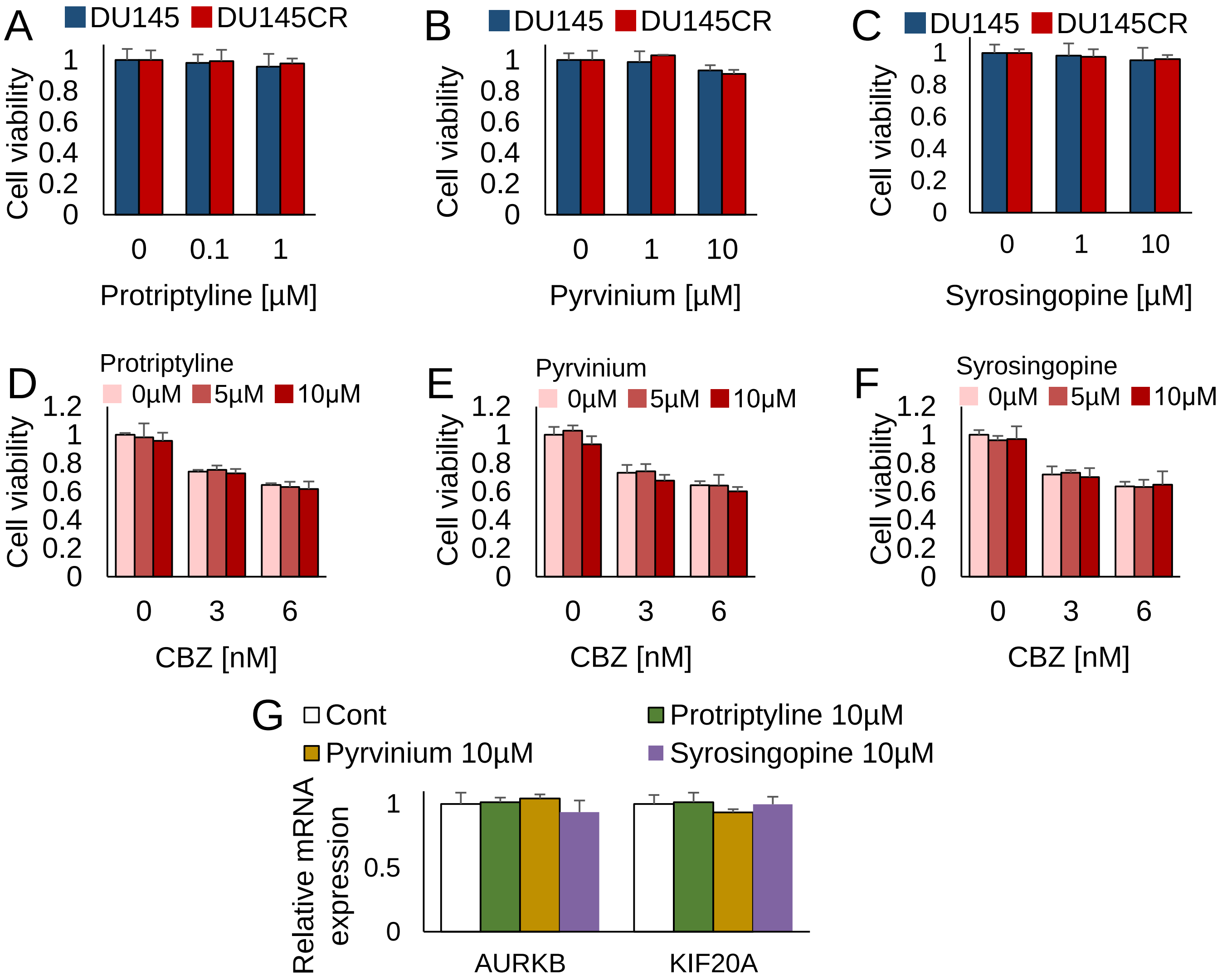

Supplementary Figure 2. Combined administration of PZD and CBZ for DU145.

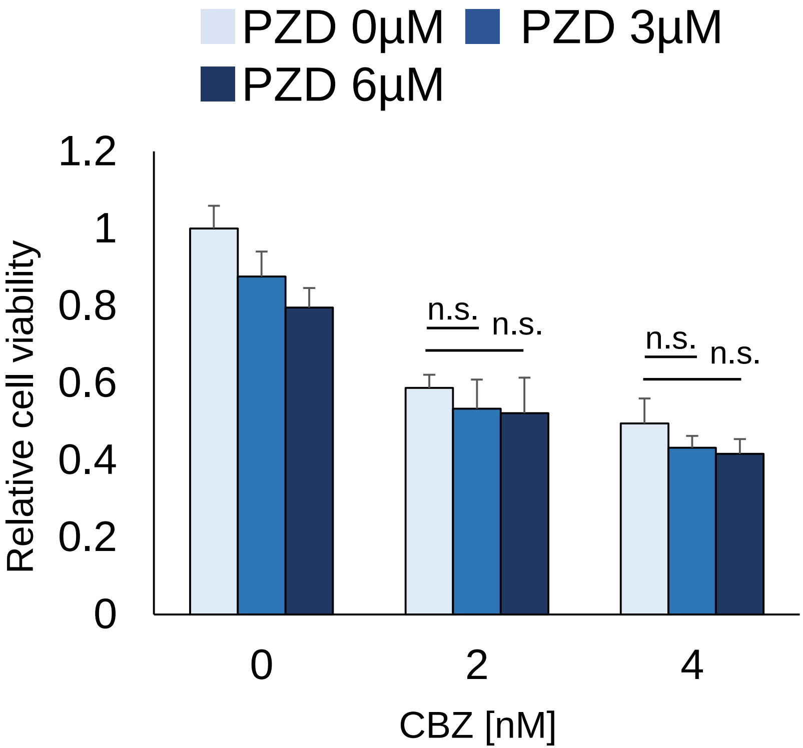

Supplementary Figure 3. Anti-tumor effect of PZD for PC3CR.

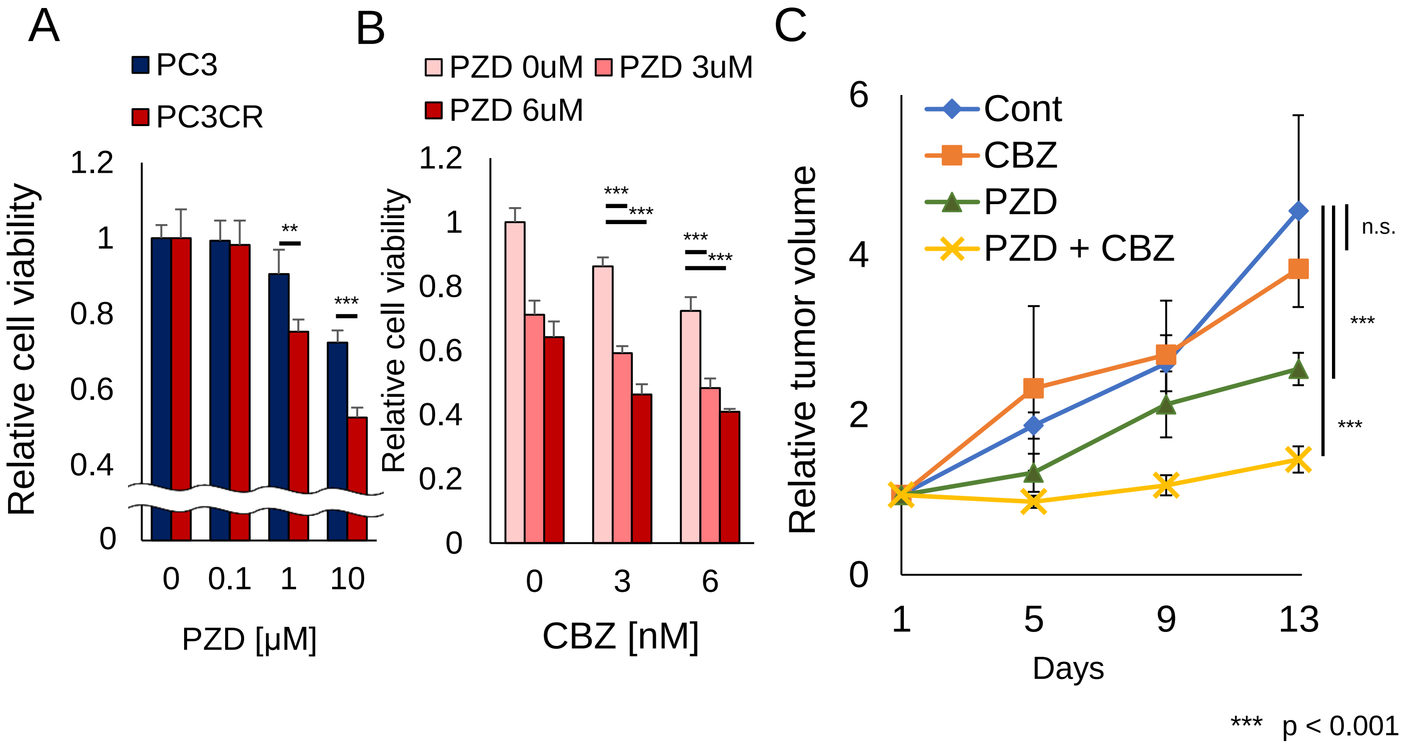

Supplementary Figure 4. Anti-tumor effect of PZD for AR positive CBZ-resistant prostate cancer cells.

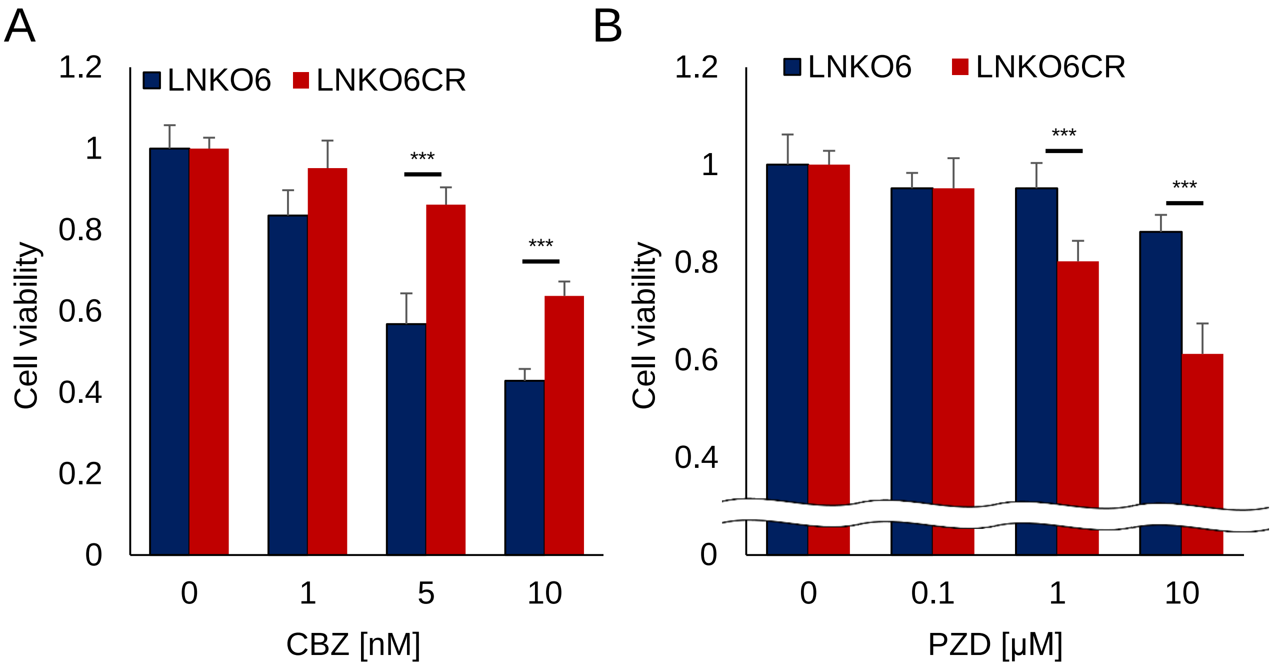

Supplementary Figure 5. Ki67 and TUNNEL staining in DU145CR xenograft tumors.

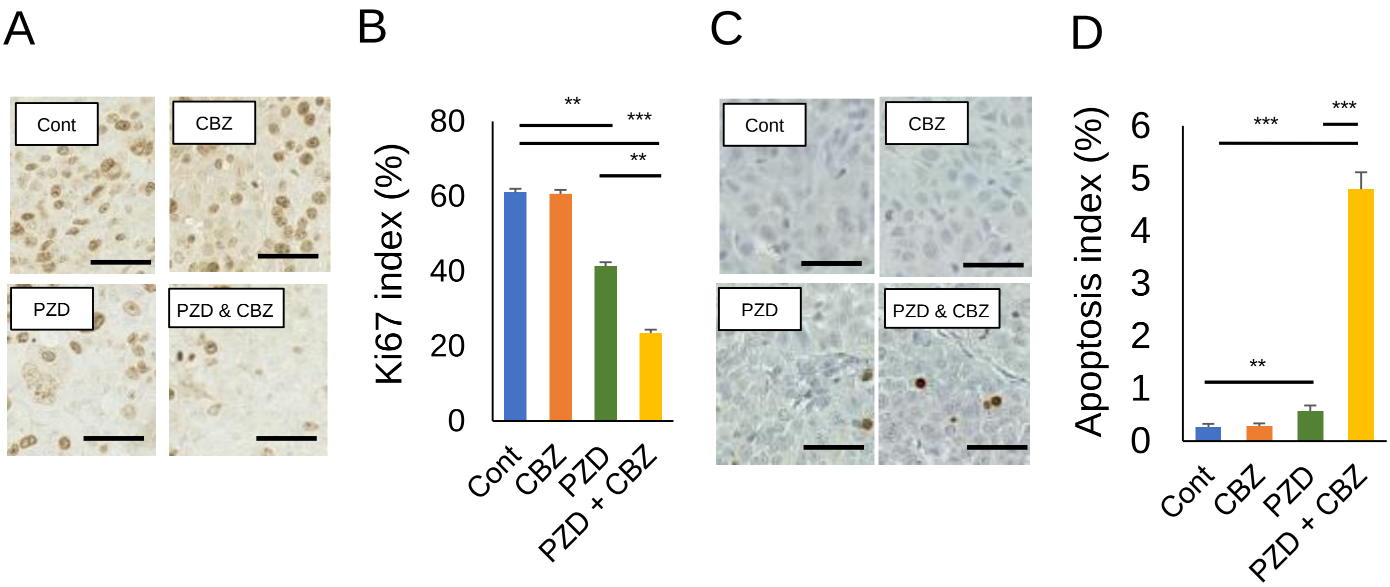

Supplementary Figure 6. Up-regulation of AURKB and KIF20A in PC3CR.

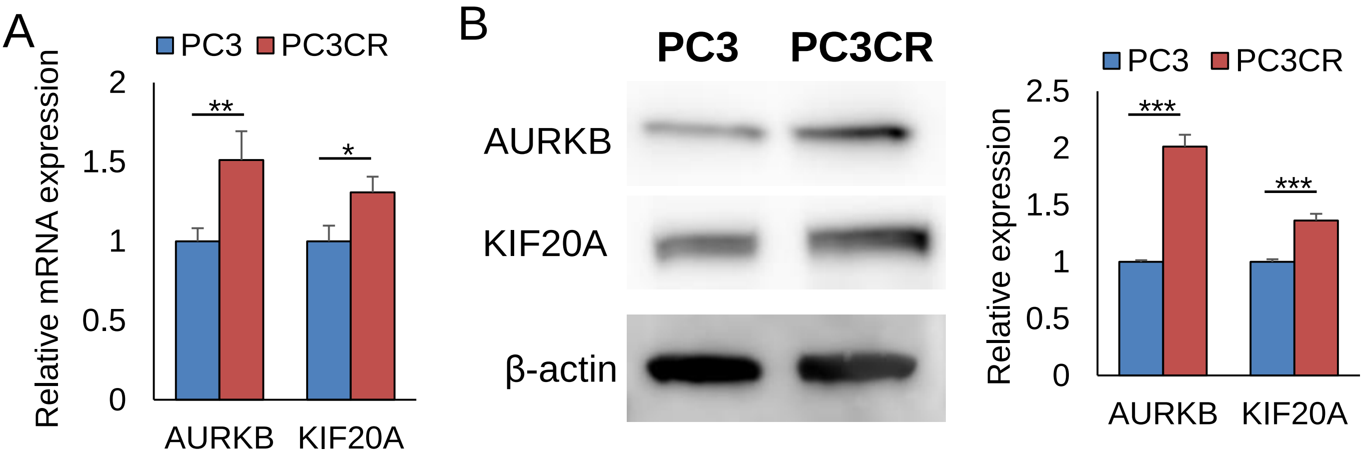

*** p < 0.001
** p < 0.01
* p < 0.05

Supplementary Figure 7. AURKB / KIF20A expression in DU145 / DU145CR xenograft model

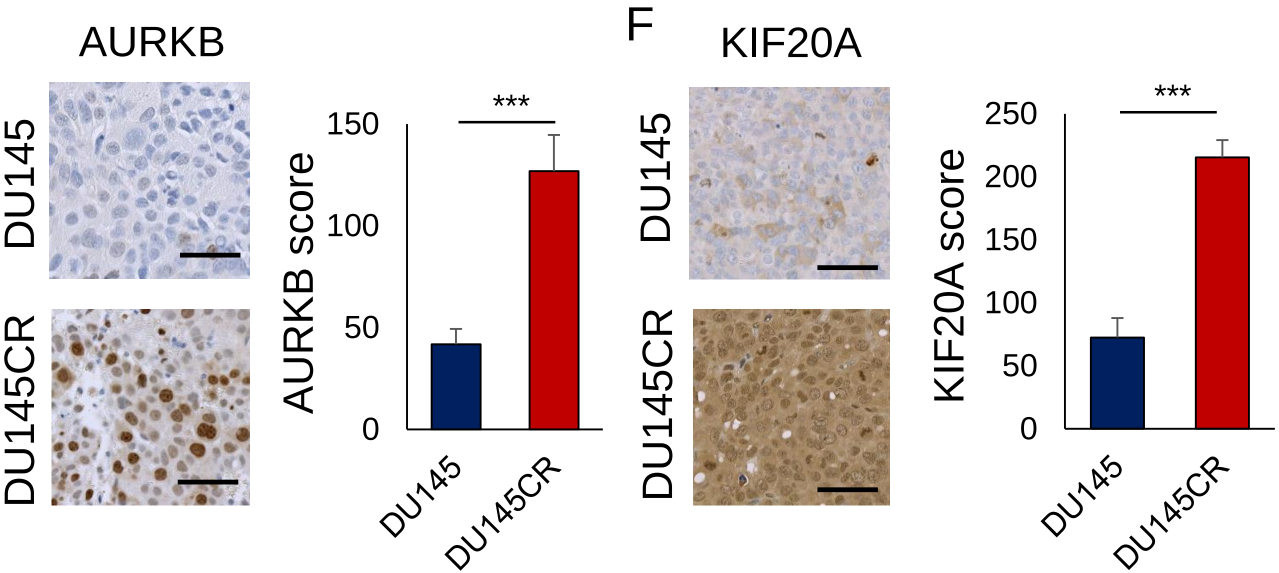

Supplementary Figure 8. Flow-cytometry of DU145CR cells.

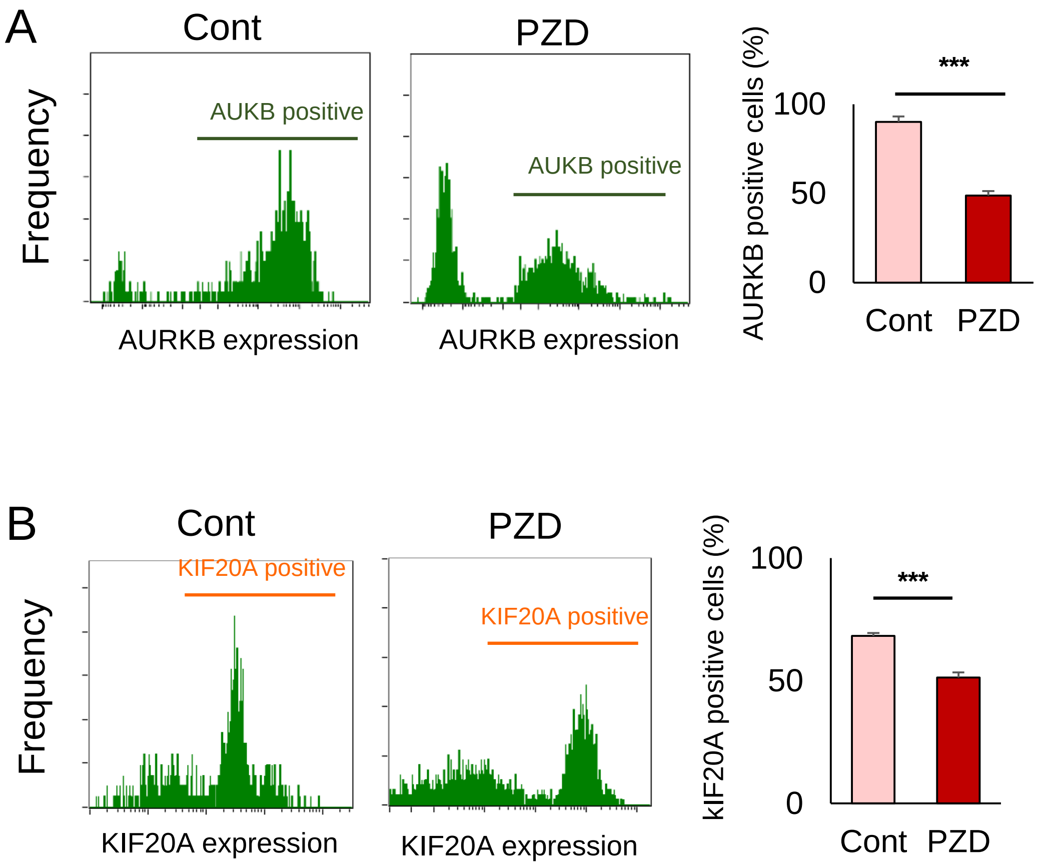

Supplementary Figure 9. Antitumor effect of AURKB or KIF20A inhibition for PC3CR.

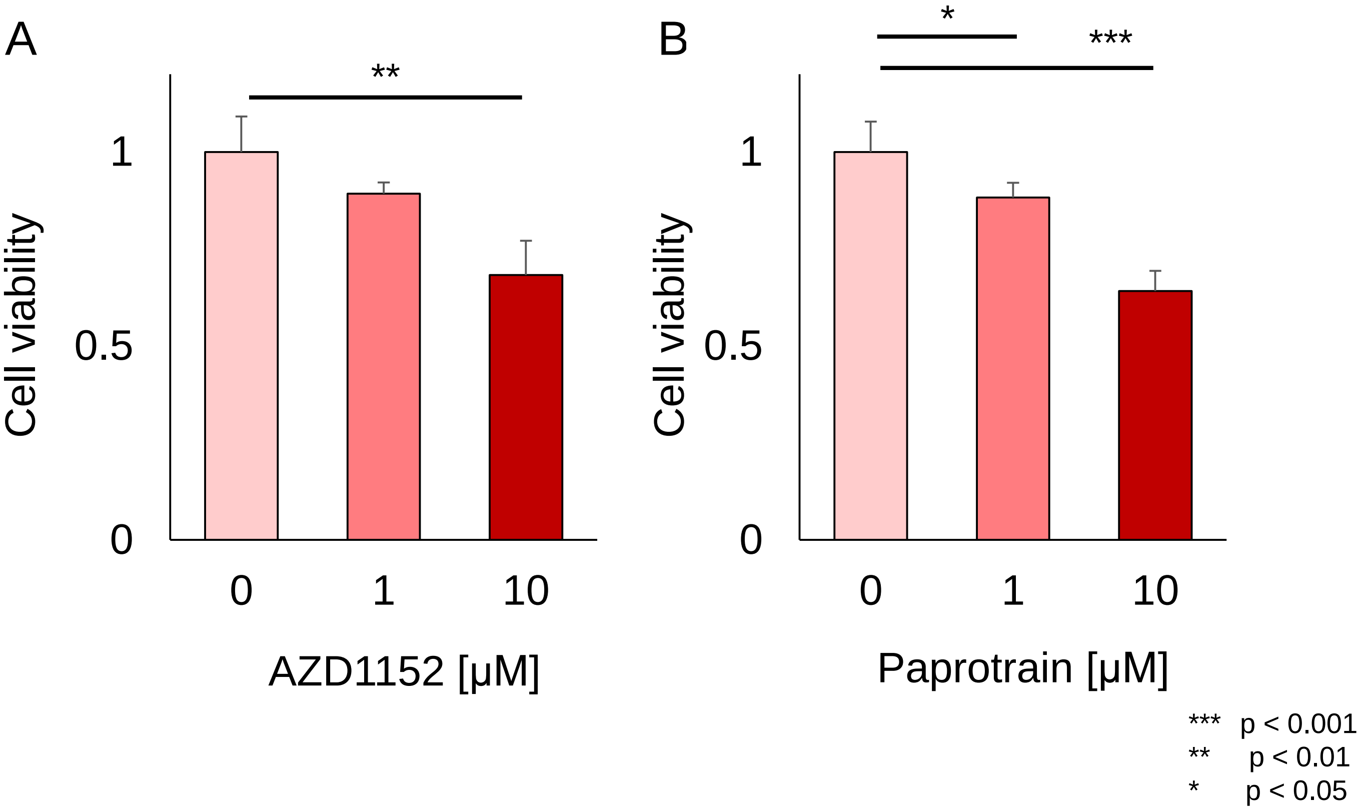

Supplementary Figure 10. Contribution of AURKB / KIF20A overexpression for CBZ resistance in prostate cancer cells.

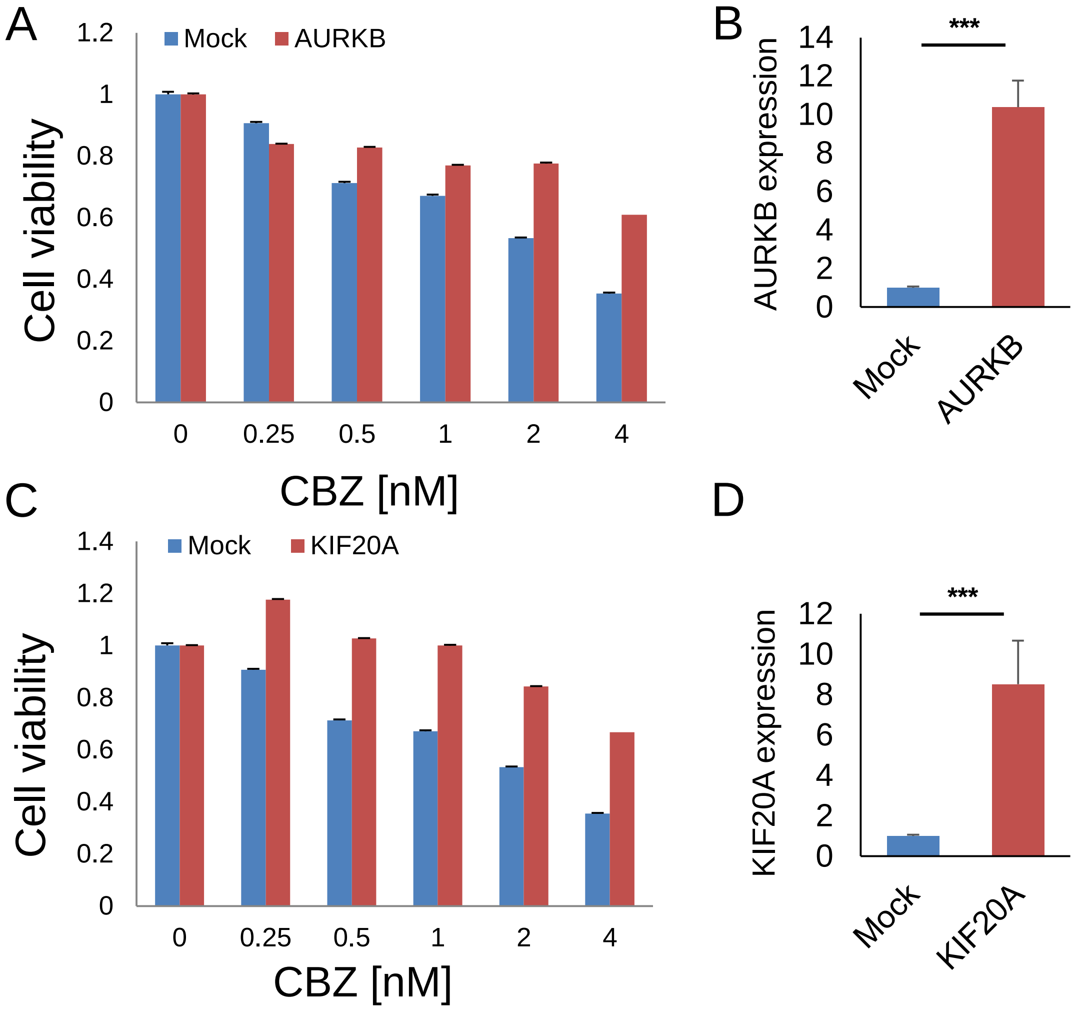

Supplementary Figure 11. AURKB and KIF20A expression in CBZ-resistant CRPC tissue.

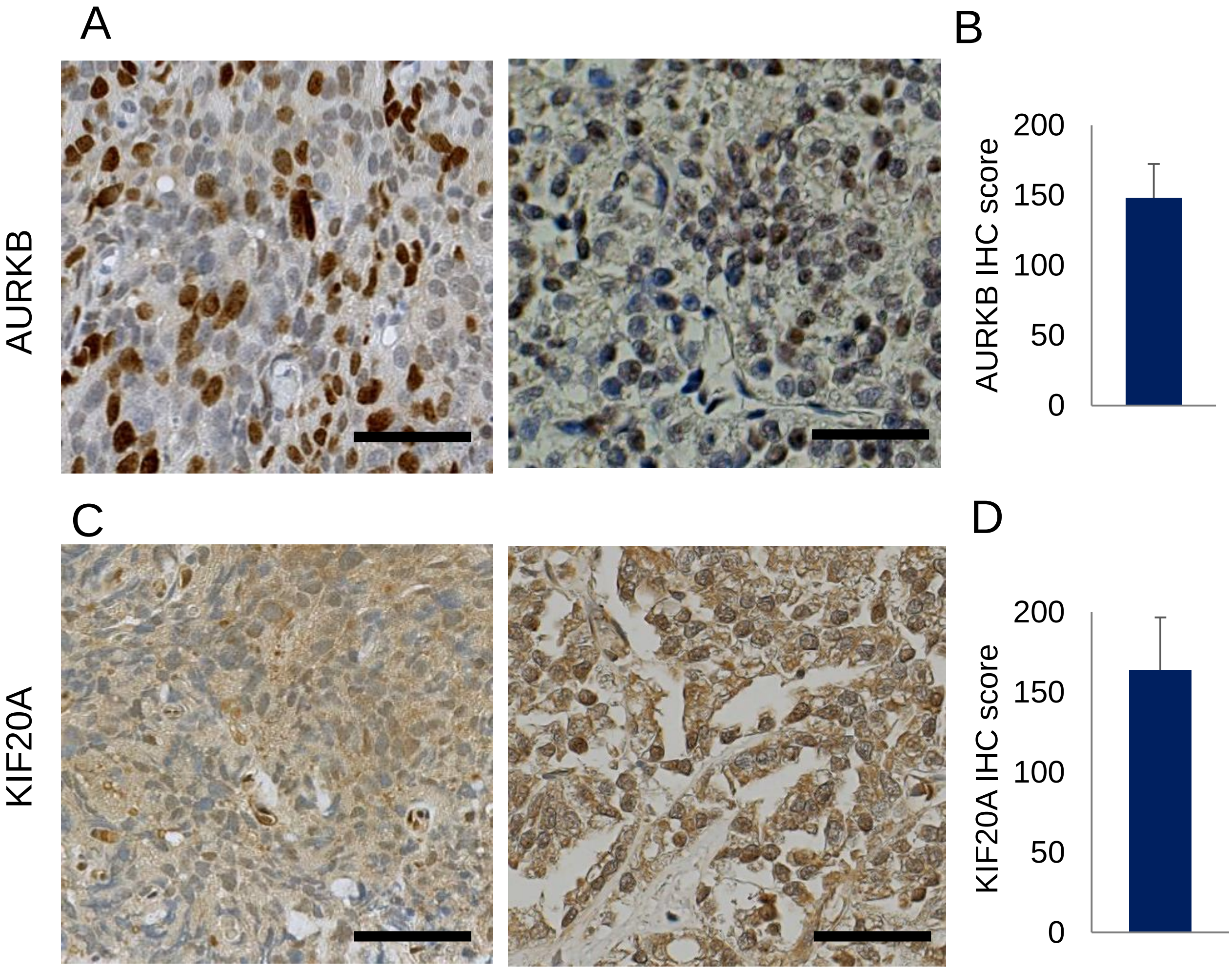

Supplementary Figure 12. Schematic representation of how pimozide exert an anti-tumor effect on cabazitaxel-resistant prostate cancer.

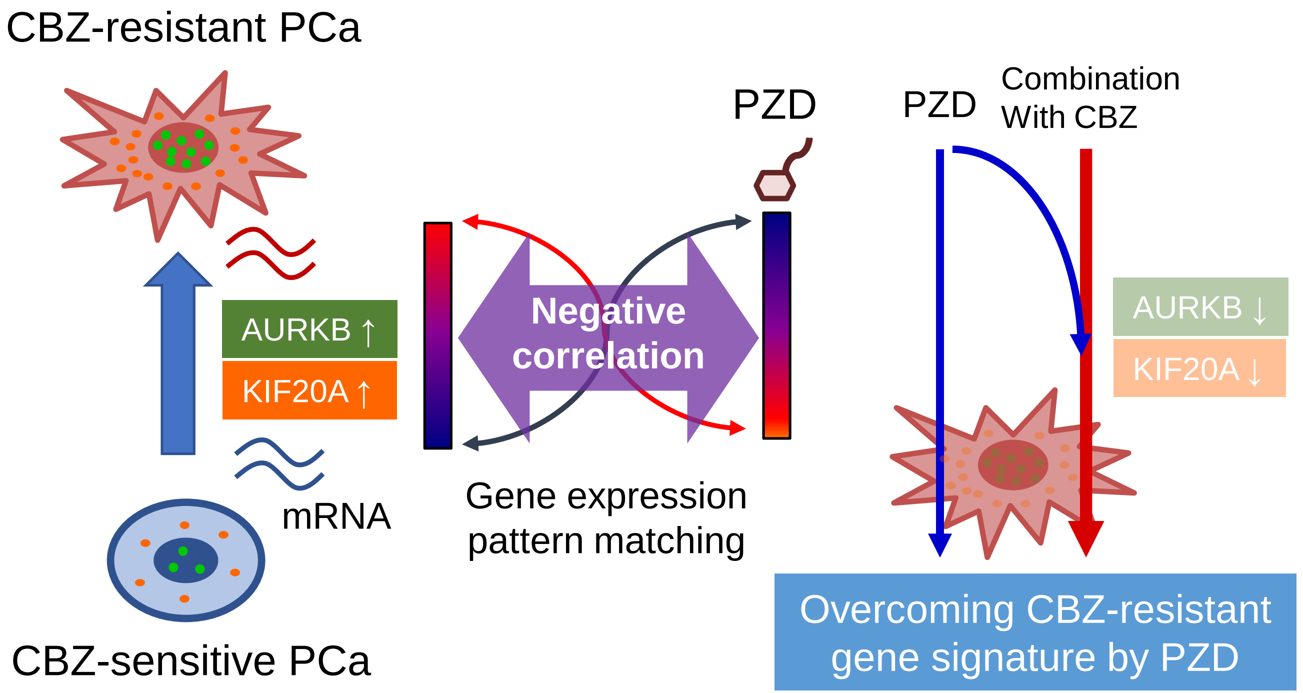

Supplement: Supplementary file 2 — Supplementary Figures [file 41391_2021_426_MOESM2_ESM.pdf]
