# Supplementary material for: Discovery of a new candidate drug to overcome cabazitaxel-resistant gene signature in castration-resistant prostate cancer by in silico screening
Source: Prostate Cancer Prostatic Dis. 2021 Sep 30;26(1):59–66. doi: 10.1038/s41391-021-00426-0 (PMC10023558; doi:10.1038/s41391-021-00426-0)
Supplement: Supplementary file 3 — Supplementary Table 1 [file 41391_2021_426_MOESM3_ESM.docx]

|  | Age  (y/o) | PSA at  Diagnosis (ng/mL) | PSA at  biopsy (ng/mL) | Site of  biopsy | Histology | Metastatic site | No. of  CBZ  cycles | Prior Tx | PFS of CBZ  (weeks) |
| --- | --- | --- | --- | --- | --- | --- | --- | --- | --- |
| Case 1 | 76 | 8.35 | 87.05 | Prostate | AdenoCa  GS 5+5 | Lymph nodes,  Bones, Liver | 6 | DOC | 25 |
| Case 2 | 70 | 636 | 700.42 | Liver | AdenoCa GS 5+4 | Liver, Bones | 10 | Ra223, DOC | 35 |
| Case 3 | 68 | 1200 | >1000 | Dura | AdenoCa  with NED | Dura, Bones,  Liver | 3 | ABI, ENZ,  DOC, Ra223 | 5 |

Supplementary Table 1. Patients’ characteristics

Tx; Treatment, AdenoCa; Adeno carcinoma, GS; Gleason score, NED; Neuroendocrine differentiation, DOC; Docetaxel, Ra223; Radium-223 chloride, ABI; Abiraterone, ENZ; Enzalutamide
