# Supplementary material for: Discovery of a new candidate drug to overcome cabazitaxel-resistant gene signature in castration-resistant prostate cancer by in silico screening
Source: Prostate Cancer Prostatic Dis. 2021 Sep 30;26(1):59–66. doi: 10.1038/s41391-021-00426-0 (PMC10023558; doi:10.1038/s41391-021-00426-0)
Supplement: Supplementary file 4 — Supplementary Table 2 [file 41391_2021_426_MOESM4_ESM.docx]

Supplementary Table 2. Candidate compounds to overcome cabazitaxel resistance in castration-resistant prostate cancer.

| Candidate  compound | Mean | Enrichment | P value | Specificity |
| --- | --- | --- | --- | --- |
| Protriptyline | −0.761 | −0.877 | 0.0005 | 0 |
| MS-275 | -0.758 | −0.966 | 0.00251 | 0.0988 |
| Syrosingopine | −0.754 | −0.816 | 0.0206 | 0.0206 |
| Norcyclobnzaprine | −0.732 | −0.905 | 0.00002 | 0.0078 |
| 0297417-0002B | −0.722 | −0.932 | 0.0005 | 0.0328 |
| Pyrvinium | −0.707 | −0.775 | 0.00024 | 0.0206 |
| Bepridil | −0.681 | −0.868 | 0.0006 | 0.0152 |
| Menadione | −0.677 | −0.902 | 0.01899 | 0.046 |
| Propafenone | −0.673 | −0.833 | 0.00145 | 0.0124 |
| Mefloquine | −0.667 | −0.735 | 0.0023 | 0.0648 |
| Pimozide | −0.647 | −0.773 | 0.00539 | 0.0354 |
| Prestwick-559 | −0.647 | −0.882 | 0.00328 | 0.0061 |
| Vorinostat | −0.643 | −0.67 | 0 | 0.1327 |
| Prenylamine | −0.633 | −0.729 | 0.01098 | 0.0734 |
| Thioridazine | −0.627 | −0.581 | 0 | 0.0645 |
| Chrysin | −0.612 | −0.773 | 0.00539 | 0.0354 |
| Trichostatin A | −0.602 | −0.58 | 0 | 0.0792 |
