# Supplementary material for: Discovery of a new candidate drug to overcome cabazitaxel-resistant gene signature in castration-resistant prostate cancer by in silico screening
Source: Prostate Cancer Prostatic Dis. 2021 Sep 30;26(1):59–66. doi: 10.1038/s41391-021-00426-0 (PMC10023558; doi:10.1038/s41391-021-00426-0)
Supplement: Supplementary file 5 — Supplementary Methods [file 41391_2021_426_MOESM5_ESM.docx]

Supplementary Methods: Animal studies in compliance with the ARRIVE guidelines

| Ethical statement | All the procedures were approved by the Institutional Review Board of Keio University School of Medicine. |
| --- | --- |
| Study design | Number of experimental groups: upon subcutaneous injection of human　cancer cell lines, mice were divided into 4 cohorts of 8 mice: an untreated control group or three treatment groups treated with intraperitoneal CBZ only (10 mg/kg), peroral PZD only (7.5 mg/kg/day), or intraperitoneal CBZ (10 mg/kg) combined with peroral PZD (7.5 mg/kg/day).  Experimental unit: 6-week-old male athymic nude BALB-C mice. |
| Experimental procedures | Human prostate cancer cell lines injection: 2.0×10^6^ cells (DU145 or DU145CR) in 100uL Matrigel were subcutaneously injected into the flanks of mice. Treatment: when tumor volume reached around 100 mm3, mice were randomly divided into 4 cohorts and treated as follows: control cohort: 0.2 ml of saline i.p. day 1, 0.2 ml of saline p.o. day 1-13. CBZ cohort: CBZ (10 mg/kg) i,p. day 1, 0.2 ml of saline p.o. day 1-13. PZD cohort: 0.2 ml of saline i.p. day 1, PZD (7.5 mg/kg/day) p.o. day 1-13. CBZ & PZD cohort: CBZ (10 mg/kg) i,p. day 1, PZD (7.5 mg/kg/day) p.o. day 1-13.  Weighing: body weight and tumor size were monitored before and after treatment, every 2-3 days. Euthanasia: at the end of the experiments, mice were anaesthetized with sevoflurane and euthanized bycervical dislocation. |
| Experimental animals | 5- to 7-week-old male athymic nude BALB-C mice, body weight 15-25g. |
| Housing and husbandry | Facility: standard facility, rooms with automatic systems of temperature, humidity and light regulation (temperature: 20-24°C; dark/light cycle: 12/12h; humidity: 60±5%). Cage: plastic, sterile, with filter. Cage companions, max 4 animals/cage. Bedding material: high adsorbing bedding material without dust, changed every week. Environmental enrichment was done with sterile material. |
| Sample size | ctrl n=8, CBZ n=8, PZD n=8, CBZ+PZD n=8. Sample size was calculated using G*power software. |
| Allocating animals to experimental groups | Mice were divided into 4 experimental groups after randomization. |
| Experimental outcomes | To evaluate the antitumor activity of PZD in *in vivo* models of CBZ-resistant prostate cancer. |
| Statistical methods | Statistical analyses were performed with t-test. The differences were considered significant at P<0.05. |
| Blinding | No blinding was done. |
